# PEGylated Cisplatin Nanoparticles for Treating Colorectal Cancer in a pH-Responsive Manner

**DOI:** 10.1155/2022/8023915

**Published:** 2022-08-05

**Authors:** Wei Li, Yongjun Sun, Jian Chen, Zhibin Jiang, Jinbao Yang

**Affiliations:** ^1^Department of General Surgery, The 980th Hospital of the PLA Joint Logistics Support Force (Primary Bethune International Peace Hospital of PLA), Shijiazhuang, 050000 Hebei, China; ^2^Department of Pharmacy, Hebei University of Science and Technology, Yuxiang Street 26, Shijiazhuang, Hebei 050018, China

## Abstract

Colorectal cancer (CRC) is a common malignant tumor, and its incidence ranks third and mortality rate ranks second in the world. Cisplatin cannot target CRC cells and has notable toxicity, which significantly limits its clinical application. The emerging PEGylated nanodrug delivery system can improve circulation time and enhance tumor targeting. In this study, the HA-mPEG-Cis NPs were synthesized by self-assembly, which can target CD44-positive CRC cells and dissolve the PEG hydration layer responsive to the weakly acidic tumor environment. The average hydrodynamic diameter of HA-mPEG-Cis NPs was 48 nm with the polydispersity index of 0.13. The *in vitro* cisplatin release was in a pH-responsive manner. The HA-mPEG-Cis NPs group showed the highest apoptosis rate (25.1%). The HA-mPEG-Cis NPs exhibited antitumor efficacy via the PI3K/AKT/mTOR signaling pathway. The HA-mPEG-Cis NPs showed the lowest tumor volume and weight among all the groups in CT26 cell-bearing mouse model. The HA-mPEG-Cis nanodrug delivery system not only increases the stability and circulation time but also reduces the side effects of loaded cisplatin. Overall, the *in vitro* and *in vivo* experiments confirmed the satisfied antitumor efficacy of HA-mPEG-Cis NPs. Therefore, this study provides a rational design for application of pH-responsive HA-mPEG-Cis nanodrug delivery system in the future.

## 1. Introduction

Colorectal cancer (CRC) is a common malignant tumor of digestive tract, and its incidence ranks third and mortality rate ranks second in the world [1, 2]. There are no obvious symptoms in the early stage of CRC, but as the tumor progresses, it will gradually show some changes in bowel habits such as diarrhea, blood in the stool, and other symptoms such as abdominal pain and anemia, threatening the health and life safety of patients [3]. The main treatment for CRC is multidisciplinary comprehensive treatment with surgery as the mainstay [4]. Meanwhile, it is necessary to cooperate with perioperative chemotherapy to reduce the risk of recurrence and metastasis [5]. Cisplatin (Cis) is situated as a platinum coordination compound with a square planar geometry and is one of the most effective chemotherapeutic agents that has been approved for the treatment of various malignant tumors including CRC [6]. However, cisplatin cannot target CRC cells, leading to the low accumulated concentration. Besides, the problems such as notable toxicity and multidrug resistance significantly limit the clinical application and effectiveness of cisplatin [7, 8].

The tumor microenvironment (TME), including the extracellular matrix, myofibroblasts, neuroendocrine cells, adipocytes, immune cells, as well as blood and lymphatic networks, is the internal and external environment of tumor cells during their occurrence, development, and metastasis [9]. Colorectal cancer interacts with TME to suppress or escape immune responses, thus leading to further progression [10]. The weak acidity of tumor tissue may be related to the accumulation of extracellular lactate and hypoxia [11]. Normal tissue cells provide the required energy through mitochondrial oxidation of glucose. However, due to the unique rapid growth rate and proliferation rate of tumor cells, tumor tissue supplies energy through the glycolytic pathway, known as the “Warburg effect” [12]. The high metabolic demands of tumor cells usually lead to the excretion of lactic acid by tumor cells to the outside of the cells causing a large accumulation of H^+^ in the TME, and the pH of tumor tissues is much lower than that of normal tissues [13].

**Figure 1:**
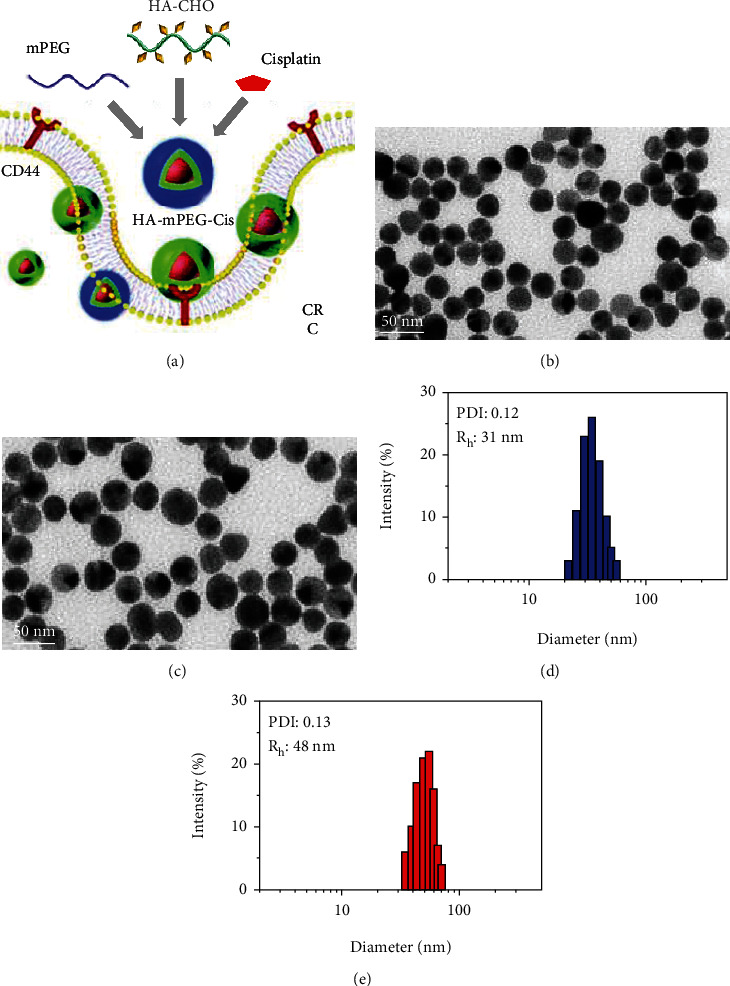
Characterization of HA-Cis and HA-mPEG-Cis NPs. (a) The schematic diagram of HA-mPEG-Cis by self-assembly targeting CD44-positive CRC cells. TEM image of (b) HA-Cis and (c) HA-mPEG-Cis NPs. Scale bar = 50 *μ*m. The size (intensity) distribution of (d) HA-Cis and (e) HA-mPEG-Cis NPs.

The emerging PEGylated nanodrug delivery system has been shown to improve drug solubility and circulation period [14, 15]. Besides, PEGylation of drug carriers can enhance phagocytosis and tumor targeting [16, 17]. To reduce the negative effects of hydrated layer on cellular uptake after PEGylation, hyaluronic acid (HA) is introduced in our PEGylated cisplatin nanoparticles (NPs). HA, the main component of the extracellular and intercellular matrix, has been approved by the Food and Drug Administration (FDA) [18]. Importantly, HA can achieve active tumor targeting through induction of CD44-mediated signaling and reduce clearance by reticuloendothelial system [19, 20]. In this study, cisplatin is grafted onto aldehyde HA (HA-Cis) through a pH-responsive imine bond, followed by grafting of methoxy polyethylene glycol amine (mPEG) onto HA-Cis (Figure 1(a)). The synthesized HA-mPEG-Cis NPs can target CD44-positive CRC cells and dissolve the PEG hydration layer responsive to the weakly acidic tumor environment.

## 2. Methods

### 2.1. Synthesis of Oxidized Sodium Hyaluronate

HA was purchased from Bloomage Biotechnology (China). NaIO_4_ and ethylene glycol were purchased from Sigma-Aldrich (USA). As previously reported [21], 1.25 g of HA was dissolved in 50 mL of water and 3.45 g NaIO_4_ was then added. The mixture was stirred in the dark for 12 h at room temperature. Then, 3.0 mL of ethylene glycol was added, and the mixture was dialyzed to obtain HA-CHO.

### 2.2. Synthesis of HA-Cis and HA-mPEG-Cis

Cisplatin, DMSO, and mPEG-NH_2_ were purchased from Sigma-Aldrich (USA). A Schiff base reaction was used to synthesize HA-Cis. Briefly, HA-CHO with the 1.31 mmol aldehyde group was dissolved in 50 mL of HAc/NaAc DMSO solution and cisplatin (0.66 mmol) was added. After stirring in the dark for 3 days, the reaction system was placed in a dialysis bag for another 3 days. To synthesize HA-mPEG-Cis, 1 g of HA-Cis and 1.2 g of mPEG-NH_2_ were added in 20 mL of DMSO with triethylamine. Then, the reaction procedure was the same as above. The obtained HA-Cis and HA-mPEG-Cis were freeze-dried and purified for further examination.

### 2.3. Characterization of HA-Cis and HA-mPEG-Cis

The powders of two NPs were dissolved in PBS for observation of the morphology by transmission electron microscope (TEM; JEOL JEM-2100Plus). The diameter and distribution of the HA-Cis and HA-mPEG-Cis NPs were examined by a Zetasizer Nano ZS90.

### 2.4. *In Vitro* Cisplatin Release

The HA-Cis and HA-mPEG-Cis solutions were placed in a dialysis bag, and four different release media with pH values of 5.0, 5.5, 6.8, and 7.4 were used for incubation in the dark at 37°C. The samples were collected at different time points, filtered, and analyzed by HPLC-FLD (Agilent Technologies, USA). The acetonitrile/0.1% formic acid in the water (30/70) was used as mobile phase, and the flow rate was set as 1.0 mL per minute.

### 2.5. Cell Culture

The murine CRC cell line CT26 was purchased from the cell bank of Shanghai (China). The CT26 cells were cultured at 37°C in 5% CO_2_ in high-glucose Dulbecco's modified Eagle's medium (DMEM) with 10% fetal bovine serum (FBS) and penicillin and streptomycin (100 U/mL).

### 2.6. *In Vitro* Cellular Uptake

The CT26 cells were seeded in a 6-well plate and cultured for 24 h. Then, the cells were treated with cisplatin, HA-Cis, or HA-mPEG-Cis (equivalent cisplatin concentration of 3 *μ*g/mL) [21]. After 1 and 4 h, the cells were collected. After washing, the cells were dissolved with HCl/HNO_3_ (3 : 1). The concentration of Pt was measured by inductive coupled plasma emission spectrometer. The cisplatin group was regarded as control, and relative concentration was calculated.

### 2.7. *In Vitro* Cytotoxicity

The CCK-8 approach (MCE Company, China) was used to evaluate the cell cytotoxicity [22]. The cells were cultured in 96-well plate for 24 h and treated with different NPs. Then, the cells were incubated for another 24 h. 10 *μ*L of CCK-8 was added, and the absorbance at 570 nm was measured by a microplate reader (Bio-Rad Company, USA).

### 2.8. Cell Apoptosis

The annexin V-FITC/PI apoptosis kit was purchased from Abcam Company (China). The procedure followed the instructions [23]. Briefly, the collected cells were resuspended in binding buffer. Then, annexin V-FITC and PI solutions were added. The reaction was kept for 15 min in the dark and analyzed by flow cytometer (BD Company, USA). Blank tube and single dye tubes were used as control.

### 2.9. Western Blot

The CT26 cells were treated with PBS, cisplatin, HA-Cis, and HA-mPEG-Cis NPs (cisplatin equivalent of 3 *μ*g/mL). The western blot was carried out as previously reported [24]. Primary antibodies including PI3K (#4249), phosphorylated PI3K (#17366), AKT (#4691), phosphorylated AKT (#4060), mTOR (#2972), phosphorylated mTOR (#2971), and *β*-actin (#3700) were purchased from Cell Signaling Technology (USA) and incubated with the proteins overnight. Corresponding secondary antibodies were purchased from Cell Signaling Technology (USA) and incubated with the proteins for another day. The molecular weight and net optical density values of the target bands were analyzed using a gel image processing system (Bio-Rad, USA).

### 2.10. Establishment of Animal Model

The male mice (about 20 g, BALB/c) were obtained from the Charles River Company (China). The animal experiment was conducted and followed the guidelines of the Animal Care and Use at the 980th Hospital of the PLA Joint Logistics Support Force and approved by the Animal Ethics Committee of 980th Hospital of the PLA Joint Logistics Support Force. The mice were treated with Cis, HA-Cis, and HA-mPEG-Cis (cisplatin equivalent dose of 5 mg/kg). The blood was taken at different time points, and the concentration of cisplatin was measured.

### 2.11. *In Vivo* Antitumor Efficiency

To establish CT26 tumor-bearing mouse model, 100 *μ*L of CT26 cells (5 × 10^5^ cells) was inoculated at the right backs of the mice [25]. When the tumor volume reached 100 mm^3^, the mice were randomly divided into 4groups including PBS, cisplatin, HA-Cis, and HA-mPEG-Cis NPs (cisplatin equivalent dose of 5 mg/kg). The tumor volume was calculated using the formula: *V* = 0.5 × *L* × *S*^2^, where *L* is the length and *S* is the width. Tumor growth curves were drawn. The body weight was recorded every two days. After 24 days, the mice were sacrificed, and the tumor was collected. The main organs (heart, lung, kidney, spleen, and liver) were collected and fixed by 4% paraformaldehyde. After dehydration, embedding in paraffin, and sectioning, HE staining was performed and histopathological changes were observed.

### 2.12. Statistical Analysis

GraphPad Prism 8 was used for data analysis and plotting figures. The data is shown as the mean ± standard deviation (SD). Two-tailed Student's *t*-test and one-way analysis of variance (ANOVA) were used for comparison. *P* < 0.05 was statistically significant.

## 3. Results and Discussion

### 3.1. Synthesis and Characterization of HA-Cis and HA-mPEG-Cis NPs

As shown in Figures 1(b) and 1(c), the HA-Cis and HA-mPEG-Cis NPs were in spherical shape and the size was uniform as well by TEM. Of note, the modification of mPEG increases the particle size. The size distribution further confirmed the results. As shown in Figures 1(d) and 1(e), the average hydrodynamic diameter of HA-Cis and HA-mPEG-Cis NPs was 31 nm and 48 nm, respectively. Moreover, the polydispersity index (PDI) of HA-Cis (0.12) and HA-mPEG-Cis NPs (0.13) showed good dispersibility (<0.20).

**Figure 2:**
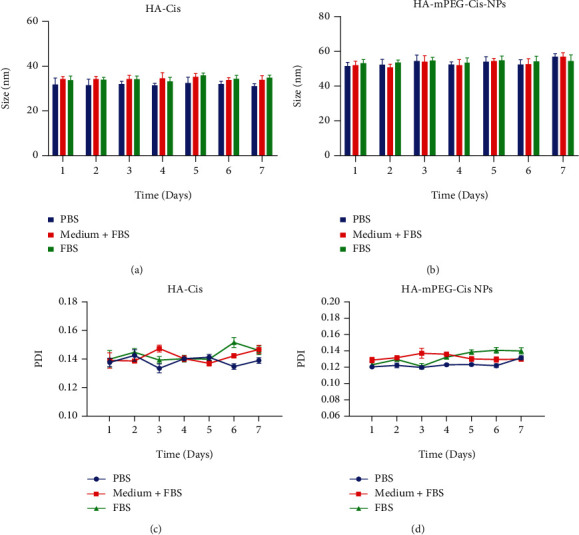
Time-dependent stability of HA-Cis and HA-mPEG-Cis NPs. Size of (a) HA-Cis and (b) HA-mPEG-Cis NPs in PBS, medium+FBS, and FBS for 7 days. The PDI of (c) HA-Cis and (d) HA-mPEG-Cis NPs in PBS, medium+FBS, and FBS for 7 days.

Furthermore, the stability of both NPs was evaluated. As shown in Figures 2(a) and 2(b), the size of HA-Cis and HA-mPEG-Cis NPs was fluctuated around 30 nm and 50 nm in PBS, FBS, and medium+FBS with an exceedingly small range (*P* > 0.05), respectively. In the meantime, the PDI of both NPs was less than 0.2 for 7 days, indicating good stability in different environments (Figures 2(c) and 2(d)). The size of both NPs was in the range of 1~100 nm, which is appropriate for NPs to pass through the vascular endothelial cells supplied for tumor [26]. Therefore, the nanodrug delivery system has the passive targeting ability by this enhanced permeability and retention (EPR) effect and achieves stronger antitumor effect [27].

### 3.2. *In Vitro* Cisplatin Release

**Figure 3:**
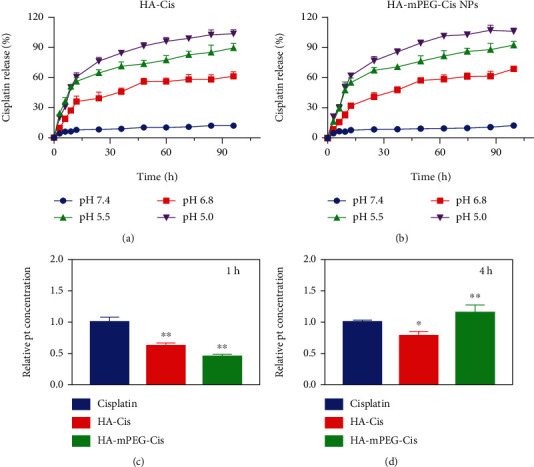
Cisplatin release and cellular uptake of HA-Cis and HA-mPEG-Cis NPs. Drug release of (a) HA-Cis and (b) HA-mPEG-Cis NPs in PBS with pH of 7.4, 6.8, 5.5, and 5.0 during 96 h. The relative Pt concentration of cisplatin, HA-Cis, and HA-mPEG-Cis NPs in CT26 cells after (c) 1 h and (d) 4 h. ^∗^*P* < 0.05 and ^∗∗^*P* < 0.01.

As shown in Figures 3(a) and 3(b), the cisplatin release from HA-Cis and HA-mPEG-Cis NPs under different pH conditions was evaluated. In the physiological conditions of blood circulation (pH = 7.4), the cisplatin release was the lowest in both NPs. With pH decreasing from 6.8 (tumor microenvironment) to 5.5 (endosomal environment) to 5.0 (lysosomal environment), the cisplatin release increased significantly. The results indicated the pH-responsive drug release of HA-Cis and HA-mPEG-Cis NPs. Importantly, the modification of mPEG did not affect the pH-dependent drug release behavior.

The inevitable toxic side effects of cisplatin on the normal tissues and cells make the patients intolerable to the chemotherapy [28]. Our cisplatin delivery strategy is based on the pH-responsive release. Once entering the in vivo environment (pH = 7.4), the significantly low drug release ensures the minimal effect of cisplatin on the normal tissues. However, when the NPs arrive the tumor site, the acidic tumor environment provokes the disintegration and significantly promotes drug release. Moreover, the release can be further enhanced in the endosome and lysosome inside the tumor cells.

### 3.3. *In Vitro* Cellular Uptake

The cellular uptake experiment was performed to evaluate the targeting ability. As shown in Figures 3(c) and 3(d), free cisplatin showed the highest accumulation in the CRC cells compared with HA-Cis and HA-mPEG-Cis NPs at 1 h. However, the HA-mPEG-Cis group showed increased cellular uptake than free cisplatin and HA-Cis at 4 h. Of note, the cellular uptake of HA-Cis and HA-mPEG-Cis NPs was enhanced at 4 h compared with 1 h, indicating a time-dependent cellular uptake especially the HA-mPEG-Cis NPs. The phenomenon in our experiment was consistent with the accepted opinion that nanodrug delivery system can prolong the circulation time and reach the tumor site later than free drug [29]. The enhanced cellular uptake may result from the HA's targeting recognition of CD44 expressed in the CRC cells [30].

### 3.4. *In Vitro* Cytotoxicity

**Figure 4:**
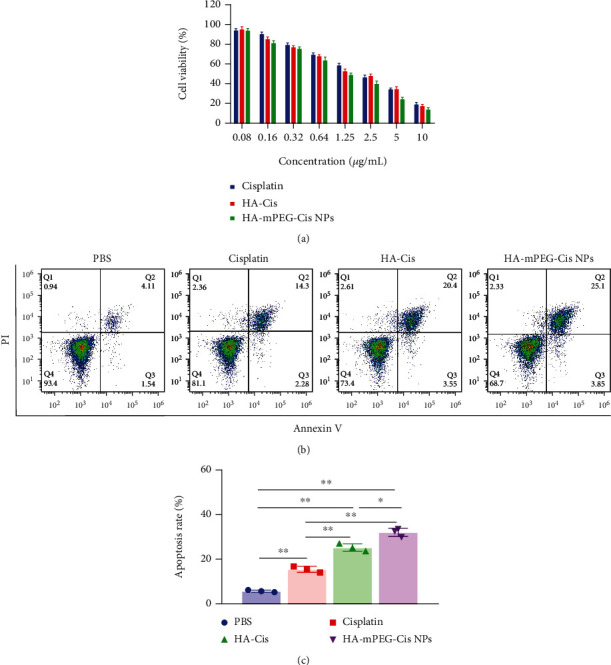
*In vitro* cytotoxicity and cell apoptosis. (a) Cell viability of cisplatin, HA-Cis, and HA-mPEG-Cis NPs in CT26 cells. (b) The cell apoptosis of CT26 cells treated with PBS, cisplatin, HA-Cis, and HA-mPEG-Cis NPs (cisplatin equivalent of 3 *μ*g/mL) in CT26 cells for 24 h. (c) Calculated apoptosis rate of each group. ^∗^*P* < 0.05 and ^∗∗^*P* < 0.01.

As shown in Figure 4(a), the CCK-8 method was used to evaluate the cell cytotoxicity. All of the cells treated with cisplatin, HA-Cis, and HA-mPEG-Cis NPs exhibited significant concentration-dependent cytotoxicity. After calculation, the IC50 of cisplatin, HA-Cis, and HA-mPEG-Cis NPs was 2.0 *μ*g/mL, 1.7 *μ*g/mL, and 1.2 *μ*g/mL, respectively. The HA-mPEG-Cis NPs showed the highest cell killing ability. Although the cell viability of the HA-Cis group seemed to be a little lower than that of the cisplatin group, there were no significant differences found between cisplatin and HA-Cis. The reason may be that the incubation time (24 h) is not long enough or the treated concentration is lower than critical micelle concentration [31].

### 3.5. *In Vitro* Cell Apoptosis

As shown in Figure 4, the cell apoptosis was studied using flow cytometry. Compared with the control group, the apoptosis rate of the cisplatin, HA-Cis, and HA-mPEG-Cis NPs groups was higher (*P* < 0.01). Importantly, the HA-mPEG-Cis NPs group showed the highest apoptosis rate (~25%), indicating a significantly enhanced antitumor efficacy. Cisplatin has been reported to have the ability of inducing apoptosis in different kinds of human cancers such as lung cancer [32], breast cancer [33], and oral squamous cell carcinoma [34]. Apoptosis plays an important role in cisplatin-induced cell death [35]. There are several mechanisms which can lead to cell apoptosis to be further explored.

### 3.6. The PI3K/AKT/mTOR Signaling Pathway

**Figure 5:**
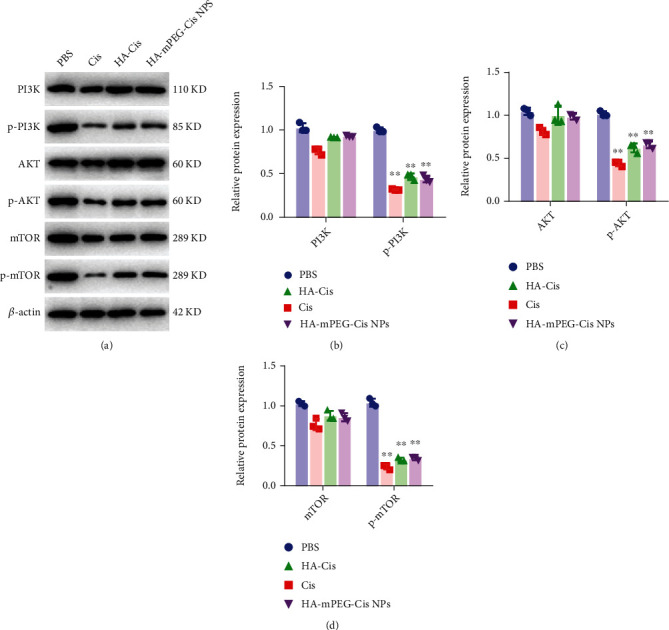
The PI3K/AKT/mTOR signaling pathway. (a) Protein expression of PI3K, phosphorylated PI3K, AKT, phosphorylated AKT, mTOR, phosphorylated mTOR, and *β*-actin by western blot in CT26 cells treated with PBS, cisplatin, HA-Cis, and HA-mPEG-Cis NPs (cisplatin equivalent of 3 *μ*g/mL). (b) Relative protein expression of PI3K and p-PI3K. (c) Relative protein expression of AKT and p-AKT. (d) Relative protein expression of mTOR and p-mTOR. ^∗∗^*P* < 0.01.

Based on the above results, we further explore the potential underlying molecular mechanisms. As shown in Figures 5(a)–5(d), the protein expressions of p-PI3K, p-AKT, and p-mTOR were significantly lower in CRC cells treated with cisplatin, HA-Cis, and HA-mPEG-Cis NPs. Meanwhile, no statistical differences were found in protein expressions of PI3K, Akt, and mTOR in four groups. Moreover, the protein expressions of p-PI3K, p-AKT, and p-mTOR in the HA-Cis and HA-mPEG-Cis groups were a little higher than those of the cisplatin group, which may be related with the modification of NPs. Therefore, the HA-mPEG-Cis NPs exhibited antitumor efficacy via the PI3K/AKT/mTOR signaling pathway. The PI3K/AKT/mTOR signaling plays an important role in carcinogenesis, acquiring drug resistance and metastatic initiation of CRCs [36]. Ma et al. reported epithelial-mesenchymal transition (EMT) and cellular apoptosis were regulated via the PI3K/AKT signaling pathway [37]. Zhang et al. found that the sensitivity of CRC cells to cisplatin was regulated by the PI3K/AKT signaling pathway [38]. Saber et al. used cisplatin and metformin nano-cubosomes to treat CRC and also found cisplatin could downregulate the expression of p-AKT and p-mTOR [39].

### 3.7. *In Vivo* Antitumor Therapeutic Effect

**Figure 6:**
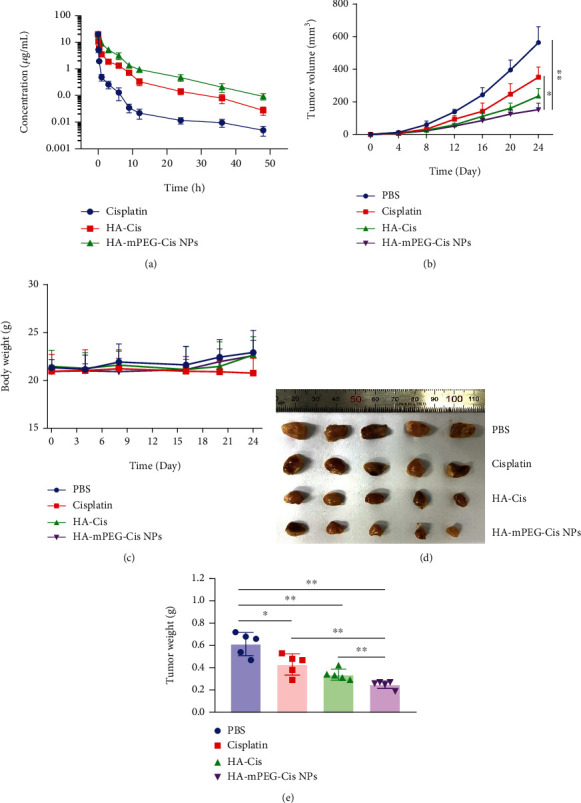
*In vivo* antitumor therapeutic effect. (a) Pharmacokinetics of cisplatin, HA-Cis, and HA-mPEG-Cis NPs (cisplatin equivalent dose of 5 mg/kg) in BALB/c mice. (b) Tumor volumes, (c) body weight, (d) tumor photographs, and (e) tumor weight of mice treated with PBS, cisplatin, HA-Cis, and HA-mPEG-Cis NPs (cisplatin equivalent dose of 5 mg/kg). ^∗^*P* < 0.05 and ^∗∗^*P* < 0.01.

As shown in Figure 6(a), HA-Cis and HA-mPEG-Cis NPs significantly prolong the circulation time in the blood compared with free drug. The results suggested that pH-responsive NPs could ensure the higher drug delivery toward tumor site by maintaining PEG modification and avoiding rapid clearance during systemic circulation. As shown in Figure 6(b), the tumor volume of HA-mPEG-Cis NPs was significantly lower than that of HA-Cis (*P* < 0.05) and PBS (*P* < 0.01). The tumor volume of HA-mPEG-Cis NPs seemed lower than HA-Cis, but there was no statistical difference as the *P* value (0.058) was more than 0.05. The body weight of mice treated with PBS and both NPs showed no decrease for 24 days, while the cisplatin group showed a trend of decrease after 16 days (Figure 6(c)). Besides, the obtained tumors of each group are shown in Figure 6(d). Consistent with the tumor volume curves, HA-mPEG-Cis NPs showed the smallest tumor and lowest tumor weight among all the groups (Figure 6(e)). Our results indicated satisfied *in vivo* antitumor efficacy of HA-mPEG-Cis NPs through pH-responsive cisplatin release.

**Figure 7:**
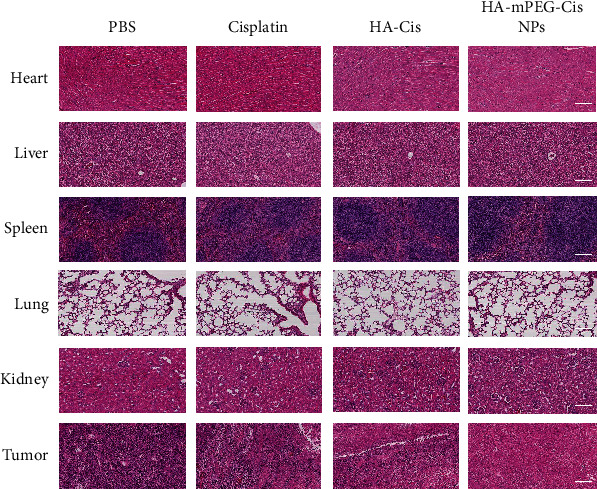
Histological change of the major organs and tumor. HE staining of the heart, liver, spleen, lung, kidney, and tumor of mice treated with PBS, cisplatin, HA-Cis, and HA-mPEG-Cis NPs (cisplatin equivalent dose of 5 mg/kg). Scale bar = 50 *μ*m.

Although the cisplatin group showed antitumor effect as well, the side effects and toxicity against normal tissues cannot be avoided. Therefore, we examined the histological change of each group (Figure 7). The tumor tissue in the PBS group showed obvious tight intertwining of blue and pink. However, the other three groups with treatment did not show this phenomenon. Of note, some damages of normal tissue such as heart and kidney were observed in the cisplatin group. As is known, the ototoxicity, nephrotoxicity, and neurotoxicity have limited the clinical application of cisplatin in treating cancers [40, 41]. Researchers have been working on how to reduce the toxicities of cisplatin. Uchino et al. used the strategy of cisplatin-incorporating polymeric micelles and reported reduced nephrotoxicity and neurotoxicity while preserving the antitumor activity [42]. Our nanodrug delivery system dramatically reduced the side effects of cisplatin while enhancing its therapeutic effect by pH-responsive strategy.

## 4. Conclusion

The synthesized HA-mPEG-Cis NPs can target CD44-positive CRC cells responsive to the weakly acidic tumor environment. The HA-mPEG-Cis nanodrug delivery system not only increases the stability and circulation time but also reduces the side effects of loaded drug through modification of HA and PEG. The *in vitro* and *in vivo* experiments confirmed the satisfied antitumor efficacy of HA-mPEG-Cis NPs. Therefore, this study provides a rational design for application of HA-mPEG-Cis nanodrug delivery system in the future.

## Data Availability

The data used to support the findings of this study are included within the article.
